# Maternal perinatal depression and child brain structure at 2-3 years in a South African birth cohort study

**DOI:** 10.1038/s41398-023-02395-5

**Published:** 2023-03-20

**Authors:** Jennifer A. Pellowski, Catherine J. Wedderburn, Nynke A. Groenewold, Annerine Roos, Sivenesi Subramoney, Nadia Hoffman, Jean-Paul Fouche, Shantanu H. Joshi, Roger P. Woods, Katherine L. Narr, Heather J. Zar, Kirsten A. Donald, Dan J. Stein

**Affiliations:** 1 Department of Behavioral and Social Sciences and International Health Institute, Brown University School of Public Health, Providence, RI USA; 2 Division of Epidemiology and Biostatistics, University of Cape Town School of Public Health and Family Medicine, Cape Town, SA South Africa; 3 Department of Paediatrics and Child Health, Red Cross War Memorial Children’s Hospital, Cape Town, South Africa; 4 Department of Clinical Research, London School of Hygiene and Tropical Medicine, London, England; 5 The Neuroscience Institute, University of Cape Town, SA Cape Town, South Africa; 6 Department of Psychiatry and Mental Health, University of Cape Town, Cape Town, SA South Africa; 7 South African Medical Research Council (SAMRC) Unit on Risk and Resilience in Mental Disorders, Department of Psychiatry, University of Cape Town, SA Cape Town, South Africa; 8 Departments of Neurology, Psychiatry and Biobehavioral Sciences, University of California Los Angeles, Los Angeles, CA USA; 9 South African Medical Research Council (SAMRC) Unit on Child and Adolescent Health, University of Cape Town, Cape Town, SA South Africa

**Keywords:** Physiology, Depression, Neuroscience

## Abstract

Maternal perinatal depression is associated with risk of adverse child developmental outcomes and differences in offspring brain structure. Evidence from low- and middle-income countries is lacking as is an investigation of antenatal, postnatal, and persistent depression in the same sample. In a South African birth cohort, we investigated the effect of antenatal and postpartum maternal depressive symptoms on offspring brain structure at 2–3 years of age. Magnetic resonance imaging was performed, extracting cortical thickness and surface areas in frontal cortex regions of interest and subcortical volumes using FreeSurfer software. Maternal depressive symptoms were measured using the Edinburgh Postpartum Depression Scale and the Beck Depression Inventory II antenatally and at 6–10 weeks, 6 months, 12 months, and 18 months postpartum and analyzed dichotomously and continuously. Linear regressions were used controlling for child age, sex, intracranial volume, maternal education, age, smoking, alcohol use and HIV. 146 children were included with 38 (37%) exposed to depressive symptoms antenatally and 44 (35%) exposed postnatally. Of these, 16 (13%) were exposed to both. Postpartum, but not antenatal, depressive symptoms were associated with smaller amygdala volumes in children (B = −74.73, *p* = 0.01). Persistent maternal depressive symptoms across pregnancy and postpartum were also independently associated with smaller amygdala volumes (B = −78.61, *p* = 0.047). Differences in amygdala volumes among children exposed to postnatal as well as persistent maternal depressive symptomatology underscore the importance of identifying women at-risk for depression during the entire perinatal period.

## Introduction

Globally, 11.9% of perinatal women experience major depressive disorder [1]. In low- and middle-income countries (LMICs) average antenatal and postpartum depression rates are much higher (19.2% antenatal, 18.7% postpartum) [1]. Maternal depression has been associated with child development outcomes [2], including socioemotional difficulties [3, 4], poor psychomotor development [5, 6], and cognitive impairment [5, 7, 8], though there are some inconsistencies in this literature [9]. In addition to associations with developmental outcomes, maternal depressive symptoms have also been associated with neurobiological alterations in offspring [2]. Neuroimaging studies have found structural differences in the brains of children whose mothers had antenatal depressive symptoms, including differences in cortical thickness [10–12], surface area [12], and subcortical volumes [13–16]. Structural differences have tended to be reported in frontal cortical regions of interest (ROIs), including the superior frontal gyrus, caudal middle frontal, pars opercularis, pars triangularis, precentral, paracentral, and frontal pole [10–12]. Subcortical volumetric differences have also been reported, particularly related to the amygdala [13–15], but also the putamen [16]. For amygdala volumes, previous studies have demonstrated mixed results, with one study finding smaller amygdala volumes at 4 years [13] and others finding enlarged amygdalae at 4.5 years [14] and 10 years [15].

The previous literature investigating the impact of maternal perinatal depression on developmental outcomes, brain growth and maturation has been limited, however, in its ability to disentangle the influence of *patterns* of depression across the pregnancy and postpartum periods. Within the child behavior literature, there is evidence for the value of taking a trajectory approach. For example, Park et al. [17] and Guyon-Harris et al. [18] found that maternal depressive symptoms that increased over the antenatal to postpartum periods were associated with more problem behaviors, poorer executive function, and greater social and emotional problems. Furthermore, Kingston et al. [19] found that persistently high depressive symptoms across the antenatal and postpartum periods were associated with greater child hyperactivity, physical aggression, and separation anxiety. Thus, patterns of maternal depression over time may have differential impacts on child behavior; however, few imaging studies have examined the impact of maternal depression patterns on underlying brain development.

Although most studies in this emerging field have tested associations with antenatal and postpartum depressive symptoms separately, there are a few exceptions. Soe et al. [20], found that maternal depressive symptoms that increased from the antenatal to postpartum period were associated with lower right frontal functional connectivity in infants at 18-months, but did not investigate the association of this pattern of depressive symptoms with brain structure. Using trajectory analyses, Zou et al. [21] reported that the trajectory consisting of persistently high levels of depressive symptoms across the antenatal and postpartum periods was associated with smaller total grey and white matter volumes in 10-year-old offspring. While they did not find significant differences in the volumes of specific subcortical structures (i.e., thalamus, amygdala, or hippocampus), these findings demonstrate the importance of capturing the longitudinal nature of perinatal depression and the differential impacts these patterns may have on brain development. In addition to greater specificity with respect to the timing of depressive symptom exposure in the antenatal and postnatal periods, most neurodevelopmental studies have been conducted in high-income countries, limiting the generalizability to LMICs which experience the highest burden of maternal perinatal depression [1]. Furthermore, neuroimaging studies around the world are often conducted with infants or older children (6–8 years or older), thus, there is a paucity of neuroimaging data capturing early childhood/toddlerhood (2–3 years).

The purpose of this study was to 1) determine the association between *antenatal* depression and cortical thickness and surface area among frontal cortex ROIs as well as subcortical volumes of children at 2–3 years, 2) determine the association between *postpartum* depression and cortical thickness and surface area among frontal cortex ROIs as well as subcortical volumes, and 3) investigate the impact of patterns of perinatal depression across the antenatal and postpartum period. To address previous limitations of the literature investigating these questions, this study utilizes data from a well-characterized South African birth cohort, which has a high prevalence of maternal depression (24.2% antenatal and 17.1% postpartum) [22–24] and undertakes neuroimaging among the understudied age group of 2–3 years of age.

## Methods and materials

**Fig. 1:**
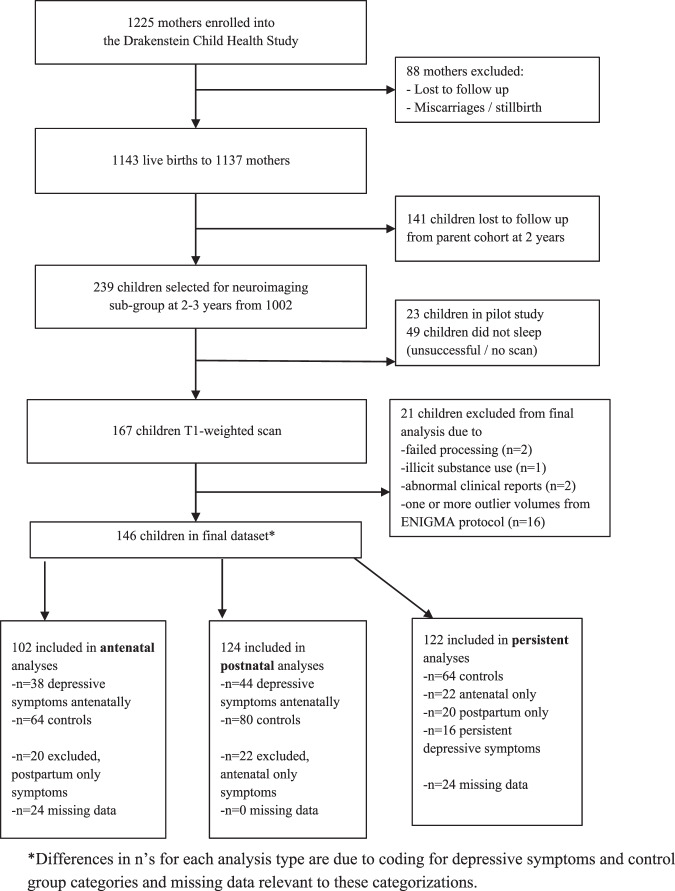
Drakenstein Child Health Study and Neuroimaging sub-study consort diagram. It shows the flow of the study design and analytic samples in the present sub-study.

The Drakenstein Child Health Study (DCHS) is a population-based birth cohort study conducted in two low socio-economic, peri-urban communities in Paarl, South Africa [22]. Between March 2012 and March 2015, the cohort enrolled 1225 pregnant women. 1137 mothers delivered 1143 live infants and this cohort has followed the mother-child dyads up to 5 years postpartum to date (see Fig. 1) [22, 25]. Biomedical, environmental, psychosocial, and demographic risk factors for child health were collected longitudinally. Ethical approval for DCHS was obtained through the Human Research Ethics Committee (HREC) of the Faculty of Health Sciences, University of Cape Town, Stellenbosch University, and the Western Cape Provincial Research committee. All participating mothers completed informed consent at enrollment and annually [22, 26].

### Neuroimaging sub-study

From the larger DCHS cohort, 239 children were invited for a neuroimaging sub-study [27]. Children were selected if they were aged 2–3 years and currently active in the DCHS cohort. Those who had received a neonatal MRI (which included sampling based on antenatal depression) were prioritized [25, 28, 29]. Additional children were selected for the 2–3-year-old neuroimaging sub-study based on several risk factor exposures (maternal HIV, and/or alcohol use) to ensure adequate representation, and a randomly selected comparison group. Children were not eligible to participate in the neuroimaging sub-study if they met any of the following exclusion criteria: 1) medical comorbidity (genetic syndrome, neurological disorder, or congenital abnormality); 2) gestation <36 weeks; 3) low Apgar score (<7 at 5 min); 4) neonatal intensive care admission; 5) maternal use of illicit drugs during pregnancy (e.g. cocaine, methamphetamines, hallucinogens, opioids, etc.); or 6) child HIV infection. Informed consent for all neuroimaging procedures was obtained from the parent.

### Demographic Characteristics

Demographic characteristics about the mother were collected during pregnancy, including maternal age, educational attainment, household monthly income, and maternal HIV status [30]. Data regarding maternal tobacco and alcohol use during and after pregnancy was also collected. The mother completed the Alcohol, Smoking, and Substance Involvement Screening Test (ASSIST) during pregnancy and postnatally, which is a self-report measure, which assesses tobacco use and alcohol use in the past three months [31]. The ASSIST has shown good reliability and validity in international, multi-site studies [32]. Tobacco and alcohol use was calculated using WHO guidelines categories of no to low risk, moderate risk, or high risk [31]. For these analyses, a score of moderate risk or high risk reported during pregnancy was categorized as fetal exposure to alcohol and/or tobacco [33]. Given the potential for under-reporting of alcohol use during pregnancy due to social desirability, women were asked again postnatally about alcohol consumption during pregnancy. These data were used to categorize any additional fetal exposures to alcohol. Maternal HIV status during pregnancy was established at enrollment by self-report and confirmed during routine HIV testing during pregnancy.

Birth outcome data were also collected, including sex, birth weight, and gestational age [34]. Birth weight was collected by trained staff using standardized hospital scales to the nearest gram. Gestational age at delivery was estimated based on antenatal ultrasound conducted in the second trimester of pregnancy. If antenatal ultrasound data was unavailable, symphysis-fundal height, recorded by trained clinical staff at enrollment, or maternal recall of last menstrual period was used.

### Perinatal depressive symptoms

All participants completed both the Edinburgh Postpartum Depression Scale (EPDS) and the Beck Depression Inventory II (BDI-II) to assess symptoms of depression among mothers during pregnancy and through 18 months postpartum. The EPDS was collected during pregnancy (28–32 weeks gestation), and at 6–10 weeks, 6 months, 12 months, and 18 months postpartum. This self-report measure asks about symptoms in the past 7 days and consists of 10 items that are rated on a scale of 0 to 3 [35, 36]. The scores are summed for a maximum value of 30. The EPDS has been validated in all three languages used (English, isiXhosa, and Afrikaans) [37–39]. We used a cut-off of greater than or equal to 13 on the EPDS indicating probable depression [35].

The BDI-II was collected during pregnancy (28–32 weeks gestation) and at 12- and 18 months postpartum. This self-report measure asks about symptoms in the past two weeks and consists of 21 items that are rated on a scale of 0 to 3 [40, 41]. The scores are summed for a maximum value of 63 and a cut-off of greater than or equal to 20 was used to indicate moderate/severe depressive symptoms [42]. This scale has been previously validated in all three languages used [43, 44]. Prescription of antidepressants was also captured during pregnancy.

### Neuroimaging procedures

Detailed protocols and procedures for this sub-study have been reported elsewhere [27]. Briefly, scanning was performed at Cape Universities Body imaging centre (CUBIC), housed at the Neuroscience Institute on the Groote Schuur Hospital campus on a research-dedicated 3 T Siemens Skyra 70 cm diameter bore whole-body MRI scanner (Siemens, Erlangen, Germany). Children were brought from home to the CUBIC at a time of day when they were due to sleep (either afternoon nap time or early evening). The team created a warm, safe, family friendly environment for the mothers and children to encourage sleep. Once the child had fallen asleep, trained study staff carefully positioned them in the scanner, with appropriate ear protection and the scan proceeded with study staff in the scan room alongside the child in order to stop the scan immediately should the child wake up and to avoid distress. The structural MRI scan protocol included a multiecho MPRAGE (MEMPRAGE) T1-weighted scan; Imaging parameters performed as follows: repetition time (TR) = 2530 ms; echo times (TE) = 1.69, 3.54, 5.39, 7.24; flip angle = 7.0°; voxel size 1.0 × 1.0 × 1.0 mm; inversion time (TI) = 1100 ms; field of view (FOV) = 224/224/176 mm, 176 slices, 1.0 mm thick; scan time: 5min21s. Standard techniques were used to optimize scanning among this age group [27].

#### Processing of MRI data

T1-weighted MR images were processed using FreeSurfer v6.0 software, an open-source software that uses automated techniques for cortical reconstruction and volumetric segmentation [45]. Processing included skull stripping, B1 bias field correction, normalization, grey-white matter segmentation, surface atlas registration and extraction, and automated cortical reconstruction per the default stream. ENIGMA protocols were used to confirm the accuracy of brain segmentation [46]. Cortical regions-of-interest (ROIs; surface area and cortical thickness) and sub-cortical volumes were extracted for this analysis. Frontal regions were selected, based on the Desikan-Killiany Atlas [47] and included caudal middle frontal, lateral orbitofrontal, medial orbitofrontal, paracentral, pars opercularis, pars orbitalis, pars triangularis, precentral, rostral middle frontal, superior frontal gyrus, and frontal pole. Subcortical volumes included the thalamus, caudate, putamen, pallidum, hippocampus, amygdala, and nucleus accumbens.

All T1-MEMPRAGE images and segmentations underwent an extensive quality check process [27]. All scans were reported by a radiologist and those with clinical abnormalities were excluded. Furthermore, scans were quality checked for movement artefact before processing. FreeSurfer outputs were then visually inspected by two assessors for errors in the segmentation of structures. Finally, images were assessed for outliers using the standard ENIGMA protocol (http://enigma.ini.usc.edu/protocols/imaging-protocols/) and these were excluded from this analysis.

### Statistical analyses

Categories of depressive symptoms were created for the antenatal and postpartum periods. For the antenatal period, the depressive symptoms category was defined as ≥20 on the BDI-II or ≥13 on the EPDS during pregnancy to capture women with higher symptom levels [48]. Controls did not meet these thresholds on either the BDI-II or EPDS during pregnancy. Participants who did not meet these thresholds during pregnancy but did meet them during the postpartum period were not included in the control group and were excluded from the antenatal analyses in order to avoid conflating antenatal and postnatal exposures. For the postpartum period, the depressive symptoms category was defined as ≥ 20 on the BDI-II or ≥ 13 on the EPDS at any time point during the postpartum period (BDI-II: 12-, 18-months postpartum; EPDS: 6–10 weeks, 6-, 12-, and 18-months postpartum). Control participants did not meet these thresholds on either the BDI-II or EPDS during any point in the postpartum period. Participants who did not meet these thresholds during the postpartum period but did meet them during the antenatal period were not included in the postpartum control group and were excluded from the postpartum analyses to avoid conflating antenatal and postnatal exposures.

Separately, to capture patterns of depressive symptoms across the antenatal and postpartum periods, a variable consisting of four categories was created, using the cut-off thresholds detailed above, such that 0=no antenatal or postpartum depressive symptoms, 1= depressive symptoms in the antenatal period only, 2=depressive symptoms in the postpartum period only, and 3=persistent depressive symptoms (i.e. depressive symptoms in both the antenatal and postpartum periods). Individuals with missing data at the antenatal time point or all postnatal time points such that patterns of depressive symptoms could not be assessed were excluded from this analysis.

Stata SE 17 was used to conduct the statistical analyses and Stata code is available on request. Demographic characteristics were compared for the depressive symptom groups (antenatal and postpartum) compared to the control groups using X^2^ tests for categorical variables and independent samples t-tests for continuous variables (significance set at *p* < 0.05, two-sided). *Minimally adjusted models* were used to examine the associations between depressive symptom categories (antenatally and postpartum) and cortical thickness and surface area in the frontal cortex ROIs using linear regressions and complete case analysis. B coefficients are reported. Associations between depressive symptom categories and subcortical volumes were also examined using linear regressions. Values (cortical thickness, surface area, and subcortical volume) for the right and left hemispheres were averaged, except where the hemisphere was tested explicitly in the subanalyses. All minimally adjusted models controlled for age at time of scan and child sex; cortical surface area and subcortical volume analyses also controlled for intracranial volume (ICV) to account for brain size. Multiple comparisons were adjusted for using the Benjamini-Hochberg false-discovery rate (FDR) correction [49]. Adjustments were made for all cortical (cortical thickness and surface area; 22 comparisons) and subcortical analyses (7 comparisons) and separately for each time point (antenatal and postnatal). Findings were considered significant at *p* < 0.05 after correction using 2 sided tests. Findings that were significant using minimally adjusted models were also tested using *fully adjusted models* adding recruitment site, maternal education, and maternal age to the minimally adjusted models. For the significant finding(s) additional analyses were conducted to further elucidate the associations. Analyses to investigate the impacts of exposure to substances (tobacco smoke and alcohol) and HIV in utero were conducted as were analyses to examine the effects by hemisphere (right and left) and child sex (female and male). Continuous analyses were also run using an average of EPDS scores across the 4 postpartum time points as sensitivity analyses. Finally, to examine patterns of depressive symptoms across pregnancy and postpartum in relation to the significant finding(s), multivariable linear regression was used with the four-category depressive symptom variable detailed above (using 3 dummy variables with no depression as the reference).

## Results

Figure 1 provides details of study flow. A total of 146 children were included in the final dataset. 102 children were included in the antenatal analyses (*N* = 38 [37%] with depressive symptoms), 124 children were included in the postpartum analyses (*N* = 44 [35%] with depressive symptoms) and 122 children were included in the persistent analyses (*N* = 16 [13%] with persistent depressive symptoms in pregnancy and postpartum. Of note, no mothers were on antidepressant medication during pregnancy.

Demographic variables were similar across depressive symptom categories (Table 1). Household monthly incomes were around R1000-5000 (USD 70-350) and most mothers had not completed secondary education (high school diploma). For fetal exposures, those in the antenatal and postpartum depressive symptom categories were more likely to be exposed to maternal smoking (X^2^ = 11.48, *p* = 0.001; X^2^ = 9.49, *p* = 0.002, respectively). 14–21% of the samples were exposed to alcohol *in utero*, but this did not differ by depressive symptom category. Finally, exposure to maternal HIV was high in this sub-study, although this was unrelated to depression status and all children remained HIV-uninfected themselves.

**Table 1:** Demographic characteristics by antenatal and postpartum depression categories.

	Antenatal Categories					Postpartum Categories				
	Depressive Symptoms		Control		Comparison	Depressive Symptoms		Control		Comparison
	*N* = 38		*N* = 64			*N* = 44		*N* = 80		
	*N*	%	*N*	%	Chi-square or Fisher’s exact test (*p* value)	*N*	%	*N*	%	Chi-square or Fisher’s exact test (*p* value)
Sex										
Female	18	47%	24	38%		22	50%	32	40%	
Male	20	53%	40	62%	0.96 (*p* = 0.33)	22	50%	48	60%	1.15 (*p* = 0.28)
Household Income										
<R1000/month	13	34%	20	31%		15	34%	22	28%	
R1000–5000/month	22	58%	40	63%		26	59%	52	65%	
>R5000/month	3	8%	4	6%	0.24 (*p* = 0.89)	3	7%	6	8%	0.59 (*p* = 0.74)
Maternal Education										
Less than secondary education	24	63%	42	66%		32	73%	51	64%	
Completed secondary education	14	37%	22	34%	0.06 (*p* = 0.80)	12	27%	29	36%	1.03 (*p* = 0.31)
Maternal HIV status										
Negative	32	84%	35	55%		25	57%	43	54%	
Positive	6	16%	29	45%	9.22 (*p* = 0.002)	19	43%	37	46%	0.11 (*p* = 0.74)
Smoking										
Moderate-high risk	15	39%	7	11%		15	34%	9	11%	
No-low risk	23	61%	57	89%	11.48 (*p* = 0.001)	29	66%	71	89%	9.49 (*p* = 0.002)
Alcohol										
Fetal exposure	8	21%	11	17%		5	14%	11	17%	
Unexposed	30	79%	52	83%	0.20 (*p* = 0.65)	30	86%	52	83%	0.17 (*p* = 0.68)
	**M**	**SD**	**M**	**SD**	**t-test (*p*** **value)**	**M**	**SD**	**M**	**SD**	**t-test (*p*** **value)**
Child age at scan (months)	34.05	1.75	33.7	1.67	–1.00 (*p* = 0.32)	34.77	1.65	33.74	1.65	−3.43 (*p* < 0.001)
Birth weight (g)	2903	613	3190	613	2.52 (*p* = 0.01)	3106	532	3140	523	−0.35 (*p* = 0.73)
Gestation at delivery (weeks)	38.52	2.95	38.77	2.31	0.46 (*p* = 0.65)	39.14	2.58	38.75	2.36	−0.85 (*p* = 0.40)
Maternal age at delivery (years)	26.24	5.04	28.08	5.35	1.71 (*p* = 0.09)	27.88	5.47	28.3	5.55	0.41 (*p* = 0.69)
Birth weight (g)	2927	100	3167	65	2.09 (*p* = 0.04)	3145.15	567.56	3125.42	566.68	−0.19 (*p* = 0.85)
Gestation at delivery (weeks)	38.67	0.46	38.77	0.29	0.20 (*p* = 0.84)	39.28	2.55	38.93	2.37	−0.78 (*p* = 0.43)
Maternal age at delivery (years)	26.38	0.81	28.25	0.63	1.81 (*p* = 0.07)	28.55	6.09	28.10	5.35	−0.44 (*p* = 0.66)

Children of mothers who experienced depressive symptoms antenatally were significantly smaller at birth (*M* = 2903 grams vs. *M* = 3190 grams, *t* = 2.52, *p* = 0.01), and children of mothers who experienced depressive symptoms postpartum were significantly older at the time of the scan (*M* = 34.77 months vs. *M* = 33.74, *t* = -3.43, *p* < 0.001).

### Antenatal depressive symptoms

Minimally adjusted models were used to identify differences in cortical thickness and cortical surface area by antenatal depressive symptom category (Table 2). Cortical thickness of the caudal middle frontal was thinner among children whose mothers experienced antenatal depression, however, this was not significant when using Benjamini-Hochberg adjusted p-values. Similarly, no significant differences were found in subcortical volumes by antenatal depressive symptom category (Table 2).

**Table 2:** Antenatal depressive symptom associations with cortical thickness, cortical surface area and subcortical volumes.

	Cortical Thickness (mm)			Cortical Surface Area (mm^2^)		
	B Coefficient	*p* value	Benjamini-Hochberg Adjusted p-value	B Coefficient	p-value	Benjamini-Hochberg Adjusted p-value
Caudal middle frontal	−0.07	0.02	0.53	5.85	0.91	1
Lateral orbitofrontal	−0.01	0.81	0.94	21.94	0.58	0.84
Medial orbitofrontal	−0.03	0.35	0.59	−2.41	0.93	0.97
Paracentral	−0.55	0.07	0.39	−35.16	0.14	0.38
Pars opercularis	−0.02	0.56	0.88	57.06	0.11	0.36
Pars orbitalis	−0.02	0.66	0.91	0.31	0.98	0.98
Pars triangularis	0.01	0.72	0.88	74.61	0.05	0.51
Precentral	−0.06	0.07	0.27	−87.46	0.19	0.46
Rostral middle frontal	−0.05	0.07	0.32	247.7	0.05	0.39
Superior frontal gyrus	−0.04	0.26	0.52	153	0.28	0.52
Frontal pole	−0.07	0.2	0.45	1.97	0.7	0.91
	**Subcortical Volume** **(mm**^**3**^**)**					
	**B Coefficient**	***p*** **value**	**Benjamini-Hochberg Adjusted p-value**			
Thalamus	99.06	0.2	0.7			
Caudate	124.46	0.14	0.98			
Putamen	33.25	0.72	0.84			
Pallidum	−4.33	0.89	0.89			
Hippocampus	56.05	0.3	0.7			
Amygdala	−26.28	0.32	0.56			
Nucleus Accumbens	−11.46	0.46	0.64			

Note: minimally adjusted linear regression models: Cortical thickness controlling for age and sex; Cortical surface area and subcortical volume controlling for age, sex, and ICV

### Postpartum depressive symptoms

Minimally adjusted models were used to identify differences in cortical thickness and cortical surface area by postpartum depressive symptom category (Table 3). No significant differences in cortical thickness or cortical surface area were found when using Benjamini-Hochberg adjusted *p* values. However, when investigating subcortical volumes by postpartum depressive symptoms (Table 3), there were significant differences in the volume of the amygdala using a minimally adjusted model, such that children of mothers who experienced depressive symptoms postpartum had significantly smaller amygdala volumes than those children of control mothers (B coeff. = −66.57, SE = 22.57, adjusted *p* = 0.03); the average amygdala size is 66.57mm^3^ smaller for children of mother who experienced depressive symptoms postpartum than for children of control mothers, all other variables held constant. This finding remained significant when using a fully adjusted model (B = −69.20, SE = 23.39, *p* = 0.004, see also Supplemental Table 2).

**Table 3:** Postpartum depressive symptom associations with cortical thickness, cortical surface area, and subcortical volumes.

	Cortical thickness (mm)			Cortical surface area (mm^2^)		
	B coefficient	*p* value	Benjamini-Hochberg adjusted *p* value	B coefficient	*p* value	Benjamini-Hochberg adjusted *p* value
Caudal middle frontal	−0.01	0.39	0.72	−40.00	0.43	0.73
Lateral orbitofrontal	−0.03	0.33	0.73	31.14	0.44	0.69
Medial orbitofrontal	−0.02	0.63	0.82	−1.18	0.96	0.96
Paracentral	−0.06	0.06	0.33	−4.63	0.85	0.94
Pars opercularis	−0.01	0.67	0.82	63.56	0.05	1.00
Pars orbitalis	−0.01	0.80	0.93	6.56	0.61	0.84
Pars triangularis	−0.02	0.50	0.73	53.82	0.08	0.35
Precentral	−0.05	0.10	0.35	−103.48	0.11	0.30
Rostral middle frontal	−0.04	0.06	0.44	113.48	0.31	0.76
Superior frontal gyrus	−0.06	0.06	0.66	9.90	0.94	0.98
Frontal pole	−0.05	0.34	0.68	−4.94	0.28	0.77
	**Subcortical volume** **(mm**^**3**^**)**					
	**B coefficient**	***p*** **value**	**Benjamini-Hochberg adjusted** ***p*** **value**			
Thalamus	7.35	0.92	0.92			
Caudate	52.46	0.50	0.88			
Putamen	13.01	0.88	1.00			
Pallidum	−4.11	0.89	1.00			
Hippocampus	−48.30	0.29	1.00			
Amygdala	−66.57	0.004	0.03			
Nucleus Accumbens	−12.61	0.38	0.89			

Note: minimally adjusted linear regression models: Cortical thickness controlling for age and sex; Cortical surface area and subcortical volume controlling for age, sex, and ICV

**Fig. 2:**
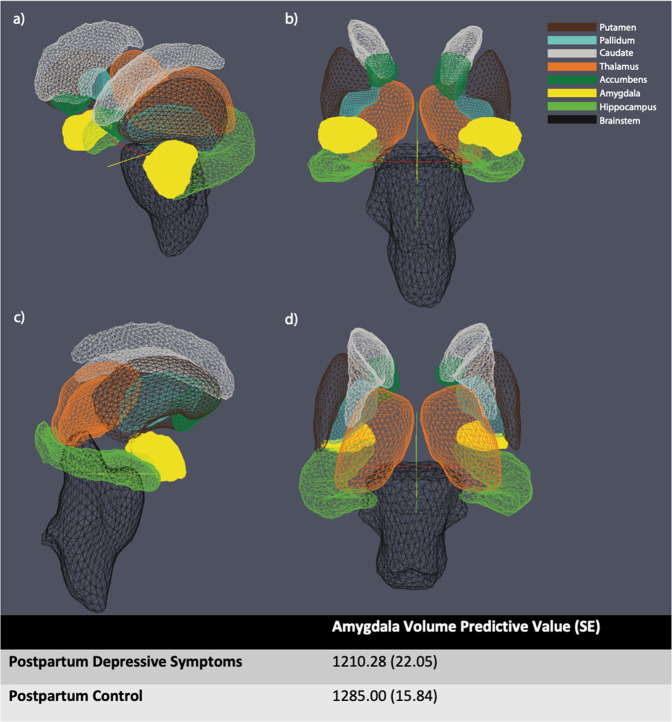
Illustration of the amygdala (in yellow) relative to other subcortical structures. The amygdala using **a** Dorsolateral, **b** anterior, **c** lateral, and **d** posterior views. Note: The amygdala volume predictive values were calculated from the amygdala regression model that controls for age, sex, ICV, maternal age, maternal education, recruitment clinic, alcohol exposure in utero, smoking exposure in utero, and maternal HIV in pregnancy.

Additional analyses were conducted to test the strength of the amygdala finding as well as to investigate important facets of the finding. To further adjust for the impact of exposure to substances and HIV *in utero*, the smoking, alcohol, and HIV exposure variables were added to the fully adjusted model; the differences in the volume of the amygdala remained (B = −74.73, SE = 28.71, *p* = 0.01; Fig. 2). Differences by hemisphere were also investigated; using fully adjusted models, postpartum maternal depressive symptoms were associated with a significantly smaller right amygdala (B = −88.55, SE = 26.07, *p* = 0.001) and left amygdala (B = −49.85, SE = 23.95, *p* = 0.04). Finally, using fully adjusted models (without child sex covariate), analyses were stratified by child sex to investigate any differences. Postpartum maternal depressive symptoms were significantly associated with smaller amygdala volumes among female children (B = −92.44, SE = 32.01, *p* = 0.006) but not male children (B = −39.93, SE = 33.84, *p* = 0.24). Continuous analyses were also conducted using the average of depressive symptoms (EPDS) across the postpartum period; the amygdala finding remained in the expected direction but was only trending on significance (B = −4.31, SE = 2.47, *p* = 0.08; Supplemental Table 1).

### Persistent depressive symptoms

The impact of persistent depressive symptoms on amygdala volume was investigated using four categories, no depressive symptoms (*N* = 64), antenatal depressive symptoms only (*N* = 22), postpartum depressive symptoms only (*N* = 20), and persistent depressive symptoms (*N* = 16; Table 4). Compared to the reference group (no depressive symptoms), children in the antenatal depressive symptoms-only group did not have significantly smaller amygdalae (B = −2.89, SE = 31.17, *p* = 0.93). Among those in the postpartum depressive symptoms-only group, children had significantly smaller amygdalae compared to the reference group (B = −71.82, SE = 33.65 *p* = 0.04), and this significant association remained significant in the fully adjusted model + exposure model (B = −79.74, SE = 35.70, *p* = 0.028. Finally, among those in the persistent depressive symptoms group, children did not have significantly smaller amygdalae compared to the reference group in the minimally adjust model but when adjusting to key covariates/exposures this association was significant (minimally adjusted B = −59.25, SE = 35.70, *p* = 0.10; fully adjusted B = −78.61, SE = 39.13, *p* = 0.047).

**Table 4:** Associations between persistent depressive symptoms across pregnancy and postpartum and amygdala volume.

		Minimally adjusted model (child age, sex, ICV)	Fully Adjusted model (child age, sex, ICV, site, maternal education, and maternal age)	Fully Adjusted model + Fetal Exposures (Smoking, Alcohol, and HIV)
	N	B Coefficient	B Coefficient	B Coefficient
No Depressive Symptoms (Reference)	64	–	–	–
Antenatal Depressive Symptoms Only	22	−2.89 (*p* = 0.93)	−10.36 (*p* = 0.75)	−24.72 *(p* = 0.45)
Postpartum Depressive Symptoms Only	20	−71.82 (*p* = 0.04)	−67.63 (*p* = 0.051)	−79.74 (*p* = 0.028)
Persistent Depressive Symptoms	16	−59.25 (*p* = 0.10)	−72.14 (*p* = 0.065)	−78.61 (*p* = 0.047)

## Discussion

This MRI study of brain structure in 2–3-year-old South African children demonstrated bilateral smaller amygdalae in children exposed to postpartum but not antenatal maternal depressive symptoms in isolation. Antenatal or postpartum depressive symptoms were not associated with structural differences in the frontal cortex (cortical thickness or surface area) nor any other subcortical volume differences when compared to control children in this study. In post-hoc analyses, the association between maternal postpartum depressive symptoms and child smaller amygdala volumes remained after adjusting for in utero alcohol, tobacco smoke, and HIV exposure. Postpartum depressive symptoms were associated with smaller amygdala volumes in both the right and left hemispheres and among female children. Finally, when examining patterns of depressive symptoms over time, children whose mothers had postpartum-only or *persistent* depressive symptoms had significant reductions in amygdala volume.

Our finding that children exposed to maternal depressive symptoms had smaller amygdalae is similar to findings from several other studies from high-income countries. In samples of newborn (2–5 weeks) and 4-year-old children in Finland, maternal depressive symptoms were associated with smaller amygdala volumes [13, 50]. However, when comparing amygdala volumes by child sex, those researchers found significantly smaller amygdalae among the male children as opposed to our study, which found smaller amygdalae among female children. The cause of these differences between studies is unclear, but may be due to social, cultural, or contextual gender differences between Finland and South Africa, that could not be controlled for. Our findings also mirror the neuroimaging literature among youth/adolescents who were diagnosed with major depressive disorder (MDD) or who are at risk for MDD due to familial depression, where smaller amygdala volumes have been reported compared to non-depressed controls [51, 52]. Further, systematic meta-analysis of this research finds smaller amygdalae among individuals who are unmedicated [53]. While the mechanisms for exposure to maternal depressive symptoms on child amygdalae development are unknown, our findings suggest that there is likely an environmental influence of exposure to a mother with depressive symptoms on brain structure [52].

In our sample, children of mothers who had postpartum only or persistent depressive symptoms across the antenatal and postpartum periods had significantly smaller amygdalae than children with no depressive symptoms. Our study adds to a small emerging literature examining patterns of maternal depressive symptoms; for example, Zou et al. [21] found persistent maternal depressive symptoms to be associated with smaller grey and white matter volumes, however, they did not find significant differences in specific subcortical volumes and they did not look at specific frontal ROIs. This study’s findings also add to the complexity to our own earlier findings within the DCHS; among infants in our cohort, antenatal depressive symptoms were associated with larger amygdala volumes among 2–6-week-old infants. This underscores the potential complexity of the persistent depressive symptom trajectory in which a larger volume was seen earlier on but changed to a loss of volume at 2–3 years. While they are preliminary, our findings regarding the amygdala underscore the importance of examining depressive symptom patterns over time to understand differential impacts on neurodevelopment.

Interestingly, we did not find differences that survived FDR correction in frontal cortical thickness or surface area, by depressive symptomatology. The only significant finding prior to correction was the cortical thickness of the caudal middle frontal by antenatal depressive symptomatology. This area has been identified in previous literature to be related to maternal depressive symptoms [12]. This lack of significant findings in our study could be due to developmental changes that do not occur until adolescence, particularly in the frontal pole. The lack of significant findings may reflect differences in context and depression phenotypes of may be due to lack of power given the sample size.

Several strengths of this study address key issues within the larger literature. This study was conducted within low socioeconomic communities within South Africa, greatly expanding the generalizability of previous similar findings in the neuroimaging literature that have been conducted in high-income countries with variable socioeconomic diversity. Furthermore, this study was conducted amongst a sample that had relatively higher incidence of moderate to severe depressive symptoms compared to previous studies [2, 23], leading to enhanced sensitivity in finding neuro-structural changes in offspring. Finally, although the prevalence of depressive symptoms was high in this context, treatment of maternal depression was very low, and only one mother was on antidepressants at the time of enrolment; this lack of treatment within the sample reduces the bias of the potential impact of in utero pharmacologic treatment on offspring brain structures [54].

Despite these clear strengths, there are a few limitations of the study that should be considered when interpreting the results of this study. In utero tobacco smoke exposure was significantly greater in the depressive symptoms groups compared to controls, which is a common phenomenon [55, 56]. While postpartum findings were still significant after controlling for in utero smoke exposure, there may still be synergistic impacts of depression and smoking exposures that are not accounted for. Furthermore, although latent maternal perinatal depressive trajectory analyses were conducted with the full Drakenstein Child Health Study cohort previously [23], the small numbers of mothers in many of the latent trajectory groups within the neuroimaging sub-sample precluded using these trajectories as the exposure variable within these analyses. Thus, a simpler analysis was conducted to account for patterns of depressive symptoms using cut-off points on the BDI and EPDS. Therefore, there may be nuanced differences on brain structures based on the timing of depressive symptoms that we were unable to capture through our current approach. Further, our continuous analyses only showed a trend toward significance, likely because of a substantial positive skew on the depressive symptom variable. Given this non-normality of our data, categorical analyses are more appropriate, however, the impact of depressive symptoms on neurodevelopment likely occurs on a continuum rather than only after reaching such cut-points [2]. Future studies should account for these subtleties when possible. Furthermore, the small sample in the persistent depressive symptom group analysis necessitates interpretation with caution, but also indicates that this is an important area for future investigation.

Another potential limitation of this study is that FreeSurfer was used for brain segmentation, but relies on the adult brain as a template. Although we used a rigorous process to ensure accuracy of brain segmentation and regional brain metrics demonstrated expected associations with child outcomes (as demonstrated in our methodological paper [25]), there may nevertheless be some measurement error still unaccounted. Additionally, the small sample of our neuroimaging study may have contributed to a lack of findings. Reproducibility is a critical issue in neuroimaging and thousands of participants are likely needed to demonstrate associations of small effect size [57, 58].The combination of similar datasets will be necessary to increase power and to advance the field. Finally, the methods utilized here cannot tease out the potential impact of genetic predisposition to severe depression that might manifest in the child as reduced amygdala volumes. However, this work contributes to our understanding of the biology of transgenerational transmission of psychopathology. Genetics and early life stress exposure interactions will continue to be a key area for future research, particularly in early childhood [59].

## Conclusions

Differences in amygdala volumes among children exposed to postnatal as well as persistent maternal depressive symptomatology underscore the importance of identifying women at-risk for depression during the entire perinatal period. While our finding of persistent depressive symptoms should be interpreted in light of the relatively small sample size, this finding indicates that further exploration of the impact of persistent exposure to depressive symptomatology across the perinatal period is an important future area of investigation. Pooling of neuroimaging study data will be an important step to ascertain the reproducibility of study findings. Further studies are also needed to determine the long-term trajectory of the amygdala, an important brain structure for emotional response, and the relevant clinical and neurodevelopmental impacts of a reduced amygdala across the life course.

## Supplementary information

Supplemental Tables
