# Supplementary material for: Maternal perinatal depression and child brain structure at 2-3 years in a South African birth cohort study
Source: Transl Psychiatry. 2023 Mar 20;13:96. doi: 10.1038/s41398-023-02395-5 (PMC10027817; doi:10.1038/s41398-023-02395-5)
Supplement: Supplementary file 1 — Supplemental Tables [file 41398_2023_2395_MOESM1_ESM.docx]

| Supplemental Table 1: Categorizations of depressive symptoms in the antenatal and postnatal periods | | | | |
| --- | --- | --- | --- | --- |
|  | **Antenatal** | | **Postpartum** | |
| Assessments | 28-32 weeks gestation (BDI-II and EPDS) | | 6-10 weeks (EPDS only) | |
|  |  |  | 6 months (EPDS only) | |
|  |  |  | 12 months (EPDS and BDI-II) | |
|  |  |  | 18 months (EPDS and BDI-II) | |
| Categorizations | **Depressive Symptoms (n= 38)** | **Control (n=64)** | **Depressive Symptoms (n=44)** | **Control (n=80)** |
| Included | ≥ 20 on BDI-II **or** ≥ 13 on EPDS at 28-32 weeks gestation | <20 on BDI-II **and** <13 on EPDS at 28-32 weeks gestation | ≥ 20 on BDI-II **or** ≥ 13 on EPDS at **any** of the postpartum time points | <20 on BDI-II **and** <13 on EPDS **all** postpartum time points |
| Excluded |  | Individuals who scored <20 on BDI-II and <13 on EPDS at 28-32 weeks gestation but who met threshold criteria during the postpartum period only (n=20) |  | Individuals who scored <20 on BDI-II and <13 on EPDS at all postpartum time points but who met threshold criteria during the antenatal period only (n=22) |
| Missing |  | n=24 |  | n=0 |

| Supplemental Table 2: Additional analyses for maternal postpartum depressive symptom associations with subcortical volumes (mm3) | | | | |
| --- | --- | --- | --- | --- |
|  | Categorical Depressive Symptoms | | Continuous Depressive Symptoms | |
|  | Fully adjusted + in utero exposures | | Minimally Adjusted | |
|  | B Coefficient | p-value | B Coefficient | p-value |
| Thalamus | 44.12 | 0.64 | 0.20 | 0.98 |
| Caudate | 78.58 | 0.44 | -2.21 | 0.78 |
| Putamen | -80.33 | 0.45 | 2.34 | 0.79 |
| Pallidum | -11.77 | 0.76 | -0.75 | 0.80 |
| Hippocampus | -36.76 | 0.50 | 1.27 | 0.80 |
| Amygdala | -74.72 | 0.01 | -4.31 | 0.08 |
| Nucleus Accumbens | -18.42 | 0.31 | -0.20 | 0.89 |
| Note: minimally adjusted models control for age, sex, and ICV; fully adjusted models control for age, sex, ICV, maternal age, maternal education, recruitment clinic, alcohol exposure in utero, smoking exposure in utero, and maternal HIV in pregnancy; | | | | |
